# A dynamic transmission model to calculate vaccination coverage needed to control COVID-19 in Germany

**DOI:** 10.1093/eurpub/ckac129.667

**Published:** 2022-10-25

**Authors:** M Waize, S Scholz, N Schmid-Küpke, J Koch, S Vygen-Bonnet, T Harder, O Wichmann

**Affiliations:** Vaccination Unit, Robert Koch-Institute, Berlin, Germany

## Abstract

**Background:**

Control of the 2019 coronavirus disease (COVID-19) pandemic in Germany and return to pre-pandemic behaviour can only be achieved through natural or vaccine-induced immunity. Sufficient vaccine capacity promotes interest in the necessary and feasible target vaccination coverage.

**Methods:**

An age and risk group stratified SEIR transmission model was used to assess the impact of vaccination coverage ranging 65%-95% for 12-59-year-olds and 90%-95% for ≥ 60-year-olds on COVID-19 incidence and intensive care unit (ICU) utilization between 01.07.2021-31.03.2022. Separate implementation of licensed vaccines allows to consider different efficacies, delivery rates and age-specific national vaccination recommendations. The analysis was conducted under different assumptions about contact behaviour during summer, reduction of daily contacts with increasing number of cases, daily vaccination uptake and the dominant variant. Data from the COVIMO study (N = 3004, data collection: 17.05.-09.06.2021) were used to define the population percentage willing to be vaccinated.

**Results:**

The COVIMO study indicates an achievable vaccination compliance rate of 83.9% among 12-59-year-olds and 94.8% among those ≥ 60-year-olds. Maximum incidence or ICU utilization during observation period decreases from 385 to 61 and 6220 to 2800, respectively, with an increase in vaccination coverage from 65% to 95% of 12-59-year-olds, 90% vaccination rate among ≥ 60-year-olds, compared to pre-pandemic reduced contact behaviour in summer and reduction of contacts as case numbers increase.

**Conclusions:**

The vaccination campaign should be continued at high intensity until at least 85% of 12-59-year-olds or 90% of ≥ 60-year-olds are fully vaccinated against COVID-19. Based on the population's willingness to be vaccinated, this goal seemed feasible.

**Key messages:**

• Mathematical modeling was used to determine an evidence-based target vaccination coverage of ≥ 85%.

• Expertise in modeling should be further strengthened.

